# Pressure-controlled microfluidics for automated single-molecule sample preparation

**DOI:** 10.1016/j.ohx.2023.e00425

**Published:** 2023-04-27

**Authors:** Anxiong Yang, Falk Nicolas Lein, Joana Weiler, Julian Drechsel, Vanessa Schumann, Felix Erichson, André Streek, Richard Börner

**Affiliations:** Laserinstitut Hochschule Mittweida, University of Applied Science Mittweida, Technikumplatz 17, 09648 Mittweida, Germany

**Keywords:** Pressure-controlled Microfluidics, Single-Molecule Spectroscopy, Single-Molecule Fluorescence Imaging, Laboratory automation

## Abstract

Sample preparation is a crucial step in single-molecule experiments and involves passivating the microfluidic sample chamber, immobilizing the molecules, and setting experimental buffer conditions. The efficiency of the experiment depends on the quality and speed of sample preparation, which is often performed manually and relies on the experience of the experimenter. This can result in inefficient use of single-molecule samples and time, especially for high-throughput applications. To address this, a pressure-controlled microfluidic system is proposed to automate single-molecule sample preparation. The hardware is based on microfluidic components from ElveFlow and is designed to be cost-effective and adaptable to various microscopy applications. The system includes a reservoir pressure adapter and a reservoir holder designed for additive manufacturing. Two flow chamber designs Ibidi µ-slide and Grace Bio-Labs HybriWell chamber are characterized, and the flow characteristics of the liquid at different volume flow rates $\dot{V}$ are simulated using CFD-simulations and compared to experimental and theoretical values. The goal of this work is to establish a straightforward and robust system for single-molecule sample preparation that can increase the efficiency of experiments and reduce the bottleneck of manual sample preparation, particularly for high-throughput applications.

## Graphical abstract

**Figure:**
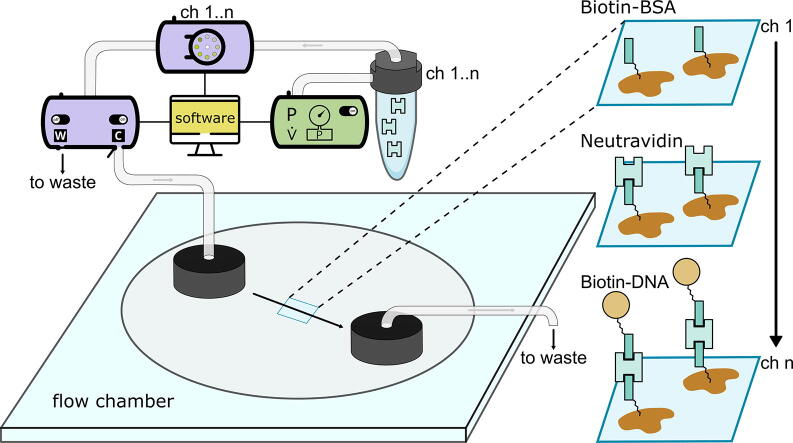

## Introduction

### Specifications table

**Table:** 

Hardware name	Microfluidics system for sample preparation automation
Subject area	• Biological sciences (*e.g.*, microbiology and biochemistry) • Chemistry and biochemistry • Medical (*e.g.*, pharmaceutical science) • Educational tools and open-source alternatives to existing infrastructure
Hardware type	• Imaging tools • Biological sample handling and preparation
Closest commercial analog	This paper utilizes commercially available hardware, which represents the current state-of-the-art technology in the field.
Open source license	GNU General Public License (GPL) 3.0
Cost of hardware	Approximate cost of hardware 7077.24 €
Source file repository	https://osf.io/r8enm/ (DOI https://doi.org/10.17605/OSF.IO/R8ENM)

## 1 Hardware in context

**Fig. 1:**
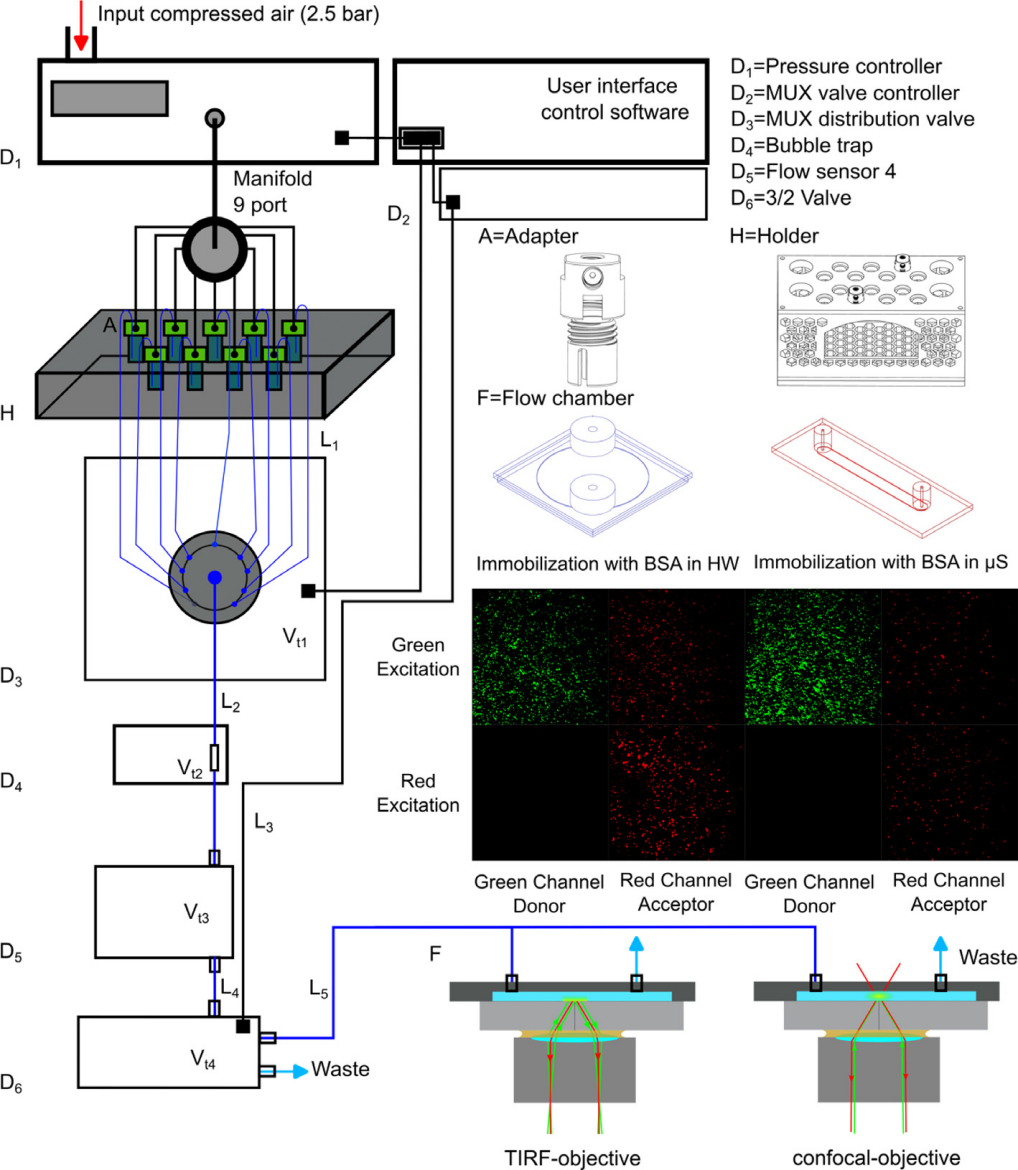
Schematic representation of the pressure-controlled microfluidic system. D_1-6_. Commercially available components of the microfluidic system (compare Table 2), A and H. Additive-manufactured components, F. Commercially available flow chambers, Grace Bio-Labs HybriWell (blue) and Ibidi µ-slide (red). Below, the false color objective TIRF image of immobilized Cy3 (green) and Cy5 (red) labelled DNA oligonucleotides after the applied BSA passivation procedure in HybriWell (HW) and in Ibidi µ-slide (µS) are shown upon 50 ms ALEX excitation for illustration. $L_{1\cdots 5}$. Length of tubes. $V_{t1\cdots 4}$ is the internal (“dead”) volume of the different components including upstream tubes (Table 3). The holder H can be combined with a buffer heater and sample heater to allow constant temperatures in the sample flow chamber.

Today, microfluidics plays a central role in numerous areas of life sciences for sample preparation and for adapting the sample to various experimental conditions [1], [2]. We use microfluidics for automated sample preparation for single-molecule experiments focusing on fluorescence microscopy [3]. A basic distinction is made between two types, total internal reflection fluorescence (TIRF) and confocal microscopy (Fig. 1) [4]. In both cases, the desired fluorescence signal is separated from the unwanted background signal, *e.g.*, autofluorescence, excitation (scattering) light etc. This is achieved either by a low penetration depth of up to a few hundred nanometers in TIRF or by a small excitation and detection volume in confocal microscopy as well as by the use of blocking filters, *e.g.*, a dichroic mirror taking advantage of the Stokes-shift [3], [5]. As such, a small sample volume is achieved, which allows the detection of single fluorescently labelled biomolecules [6], such as nucleic acids [7], [8], proteins [9], and lipids [10] to study their structure [11] and dynamics [12] beyond the ensemble average. Single-(bio)molecule detection requires various, usually time-consuming, repetitive, and error-prone preparation steps [13], [14]. Their exact execution contributes decisively to the success of the experiment [15], [16], [17].

The preparation of the sample itself and the sample chamber is commonly carried out manually by experienced researchers [18]. However, this manually done sample preparation has clear weaknesses, such as the complicated and cumbersome work steps that are time-consuming and increase the risk of contamination at every step [18], [19]. Furthermore, the reproducibility and correlation between artifacts, errors, and the method have not been well discussed in the literature, although the optimization of the protocol is considered a requirement and necessity [20]. To increase reproducibility and to save time we aim to develop an easy to apply and fully automated sample preparation procedure.

Software-controlled, automated microfluidic systems have become a versatile tool for delivering and changing buffers for fluorescence microscopy and spectroscopy applications [21]. Therein, peristaltic (e.g. Elemental Scientific, Nebraska, US) and syringe pumps (e.g. Longer Precision Pump Co., China) are used frequently and enable the delivery of volume flow rates over a wide range for a large number of channels [22], [23], [24], [25]. To deliver multiple solutions separately, multiple pumps or syringes are necessary leading to complicated and expensive designs [23]. Commercial syringe pumps load up to ten syringes, providing individual control over a wide range of channels. The limitations of syringe pumps are the fluctuation in the volume flow rate and the pressure as well as the limited delivery of very small volumes (µL) [26], [27]. The latter is achieved via microliter syringes which are not only expensive, but have usually a small total volume (1 mL) leading to frequent user interventions. The miniaturization of peristaltic pumps and their implementation on microfluidic chips has revolutionized lab-on-a-chip applications [28], but lead to complicated and expensive chip designs.

Here, we present a pressure-controlled microfluidic system that allows multi-channel µL volume applications at almost constant volume flow rates for the automation of all steps in a single-molecule fluorescence experiment: 1. the passivation of the sample chamber, 2. the immobilization of the sample and 3. the setting of the buffer and imaging conditions for the respective experiment [16]. We build on an existing commercially available system (ElveFlow Elvesys, France) and adapt it for single-molecule experiments. The advantages of the pressure-controlled device are the comparably easy and automated control of the individual sample and buffer reservoirs, the high number of available channels without the need for more pumps, and the easy and compact connection of all reservoir tubes into one final tube without the need of further manual valves. The latter reduces the total dead volume and the probability of errors due to trapped air bubbles. The system consists of a controllable compressed air sampling point that distributes the compressed air to different reservoirs, *i.e.*, micro reaction tubes (here also referred to as Eppendorf tubes), via a manifold and thus controls the volume flow from the reservoir to the microfluidic tubing system. A downstream collector brings all reservoirs together to exactly one tube, which is connected to the sample chamber. This allows injection of different buffers and sample media into the sample flow chamber one after the other in a time- and volume-controlled manner. The pressure of the compressed air is transferred to the micro reaction tubes and thus to the tubing system via an adapter which design was adapted for additive manufacturing. The applied pressure *p* controls the volume flow rate $\dot{V}$ and the respective experiment determines the number of adapters, *i.e.*, the *No.* of channels *n*, and the required pressure.

For single-molecule fluorescence microscopy, the sample chamber must be inert and free of fluorescence contamination. This is first achieved by using surfaces that can be easily cleaned with various wet chemical protocols or by using a plasma cleaner [16]. Depending on the type of microscope, simple glass coverslips (#1, BK7) for high NA objective-based TIRF and confocal microscopy or quartz coverslips for prism-based TIRF are suitable [29]. In either case, it has proven useful to enclose the sample in a (flow) chamber to protect the sample from contamination [15], [16]. For this purpose, different designs are available, of which we will describe and characterize two widely used models: a rectangular (Ibidi GmbH, Germany) and a round (HybriWell TM, Grace Bio-Labs, US) geometry [30], [31]. There have been highly integrated microfluidic chamber designs [1], however, we use single flow chambers which are commercially available, affordable, frequently used, and versatile in adapting to different experimental setups and conditions. In each case, the manufacturers guarantee that the sample chamber is free of contamination. Moreover, both chambers have been proven to allow easy replacing the microscope coverslip for single-molecule fluorescence experiments; a tedious task in former protocols [18]. The chamber volume of both chambers is in the range of µL, which is acceptable for single-molecule sample preparation. Most importantly, both chambers are easy to handle with regard to the tubing connection and thus, their replacement within the microfluidic setup. The whole system is compatible with any microfluidic inlet and outlet design, which enables fast, simple, and versatile replacement and disconnection of sample chambers.

Depending on the experiment, it must be ensured that the sample does not or only selectively interact with the chamber’s surface, *i.e.*, does not adhere to the surface (electrostatically) or react with it (chemically). This process of surface treatment is called passivation. Here, the surface is saturated with bovine serum albumin (BSA), for example, a protein that adheres to the glass surface through electrostatic interaction and prevents other biomolecules from binding. Another technique is to coat the surface with non-reactive polymers, such as polyethylene glycol (PEG) [16]. The latter, by chemically binding to the glass surface, has the advantage that the experimentally accessible range of ionic strengths, pH values, and temperatures is significantly larger than with biological passivation.

When performing an imaging experiment, *i.e*. TIRF or laser scanning microscopy (LSM), the single-molecules are usually immobilized onto the sample chamber glass surface [18]. For this purpose, functionalized passivation is used employing biotinylated molecules, *e.g.,* biotin-BSA or biotin-PEG [32], to which the sample molecules or sample reservoirs such as DNA origami [33] or lipid vesicles [10] can bind specifically, *e.g.,* using streptavidin-mediated immobilization. Subsequently, the desired experimental conditions are set by the choice of the imaging buffer to not only reach the desired sample conditions but to protect the fluorescent sample, *e.g.,* from bleaching and blinking [34]. For single-molecule fluorescence microscopy, the oxygen content of the sample has a significant impact on the experiment. Excess oxygen is responsible for photobleaching and reduces the observation time of the fluorescence-labelled single-molecule [35].

We focus on providing a pressure-controlled, cost-effective microfluidic solution for single-molecule sample preparation that is widely applicable and can be adapted by anyone to their individual experimental needs. Our approach can be adapted to existing and lab-specific protocols and can in principle be extended for any applications in microscopy which involve different scenarios: 1. an unprepared microscopy flow chamber, 2. a passivated flow chamber without sample, and 3. a ready-to-use flow chamber with sample. When used correctly, our design allows different experiments to be carried out with the same microfluidic system in succession, without the need for a separate microfluidic. We characterize two flow chamber designs, namely Ibidi µ-slide and Grace Bio-Labs HybriWell chambers concerning volume flow rates and flow velocities employing theoretical flow calculations and simulations. Further, we characterize different additive-manufactured buffer reservoir adapters regarding air tightness and *p*-$\dot{V}$-dependency within the microfluidic tubing system.

## 2 Hardware description

The compressed air, thus, pressure-controlled single-molecule sample preparation system (Fig. 1, for components, see Table 1) is based on a commercially available control system from ElveFlow (see Table 2) [36]. The system was adapted on both the hardware and software side so that it can be expanded to up to nine buffer reservoirs without additional costs to allow single-molecule sample preparation including passivation and immobilization. By combining a flow sensor, a bubble trap, and a valve system, complete automation of the preparation process is possible. The customization comprises the reservoir adapter A (parts 1 and 2) and the holder H (parts 1 and 2) as STL files for additive manufacturing as well as the computer-assisted design files (CAD, easm, eprt) for both tested flow chambers, which have been characterized herein. Moreover, it includes the software protocol sequence (sq) for the pressure-controlled microfluidics for both the PEG and the BSA passivation, including flow and pressure sensors as well as all valves.

**Table 1:** File description.

Design file name	File type	Open-source license	Location of the file
Schematic of the hardware assembly	png	GNU General Public License (GPL) 3.0	https://osf.io/sd5q8
3D-printed adapter part 1	STL	GNU General Public License (GPL) 3.0	https://osf.io/mqxhb
3D-printed adapter part 2	STL	GNU General Public License (GPL) 3.0	https://osf.io/y7zxv
3D-printed holder part 1	STL	GNU General Public License (GPL) 3.0	https://osf.io/5nj3y
3D-printed holder part 2	STL	GNU General Public License (GPL) 3.0	https://osf.io/72qrh
HybriWell chamber	easm	GNU General Public License (GPL) 3.0	https://osf.io/v5tk4
Ibidi µ-slide chamber	eprt	GNU General Public License (GPL) 3.0	https://osf.io/4t5rw
Sequence for biotin-BSA passivation and sample preparation	sq	GNU General Public License (GPL) 3.0	https://osf.io/szb25
Sequence for sample preparation after biotin-PEG passivation^1^	sq	GNU General Public License (GPL) 3.0	https://osf.io/2be4r

1 The PEG passivation, i.e., PEGylation procedure, cannot be performed within the microfluidic system and is explained in the SI.

**Fig. 2:**
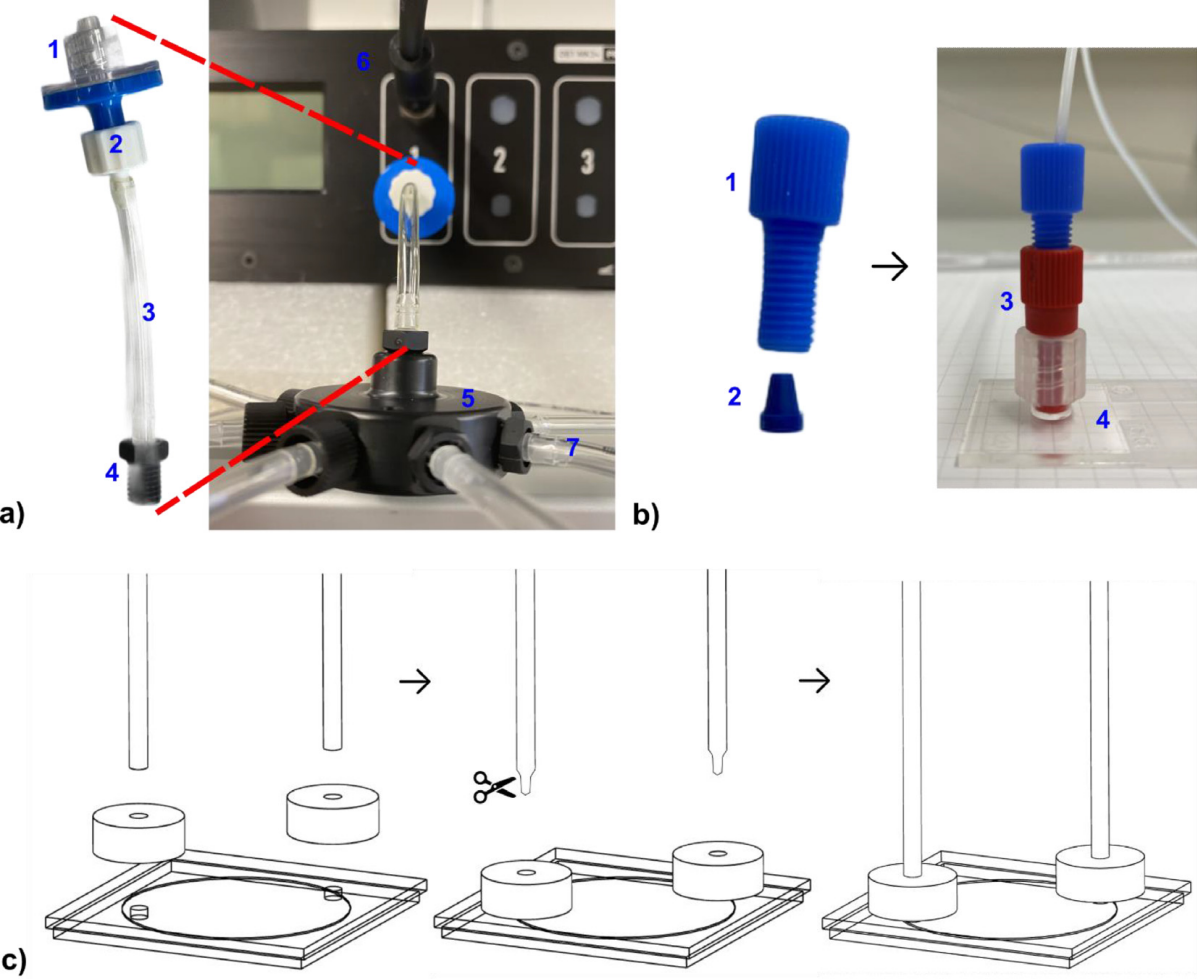
a) 1. Hydrophobic filter, 2. Male luer integral lock ring to bard, 3. 3/32“ internal diameter tube, 4. Swivel barbed adapter, 5. Nine port manifold, 6. Cable connection with flow sensor, 7. Pressure outlet to reservoir *No. n*; b) Ibidi µ-slide flow chamber with tube connection: 1. Flangeless fitting, 2. Ferrules, 3. Male luer lock adapter, 4. Ibidi µ-slide chamber; c) Grace Bio-Labs HybriWell flow chamber tubing connection. To easily connect the tubes to the press fit tubing connectors on the adhesive HybriWell chamber one needs to cut the tubes at an angle of approximately 45°. Otherwise, the press fit tubing connectors tend to eject the tubes over time.

**Fig. 3:**
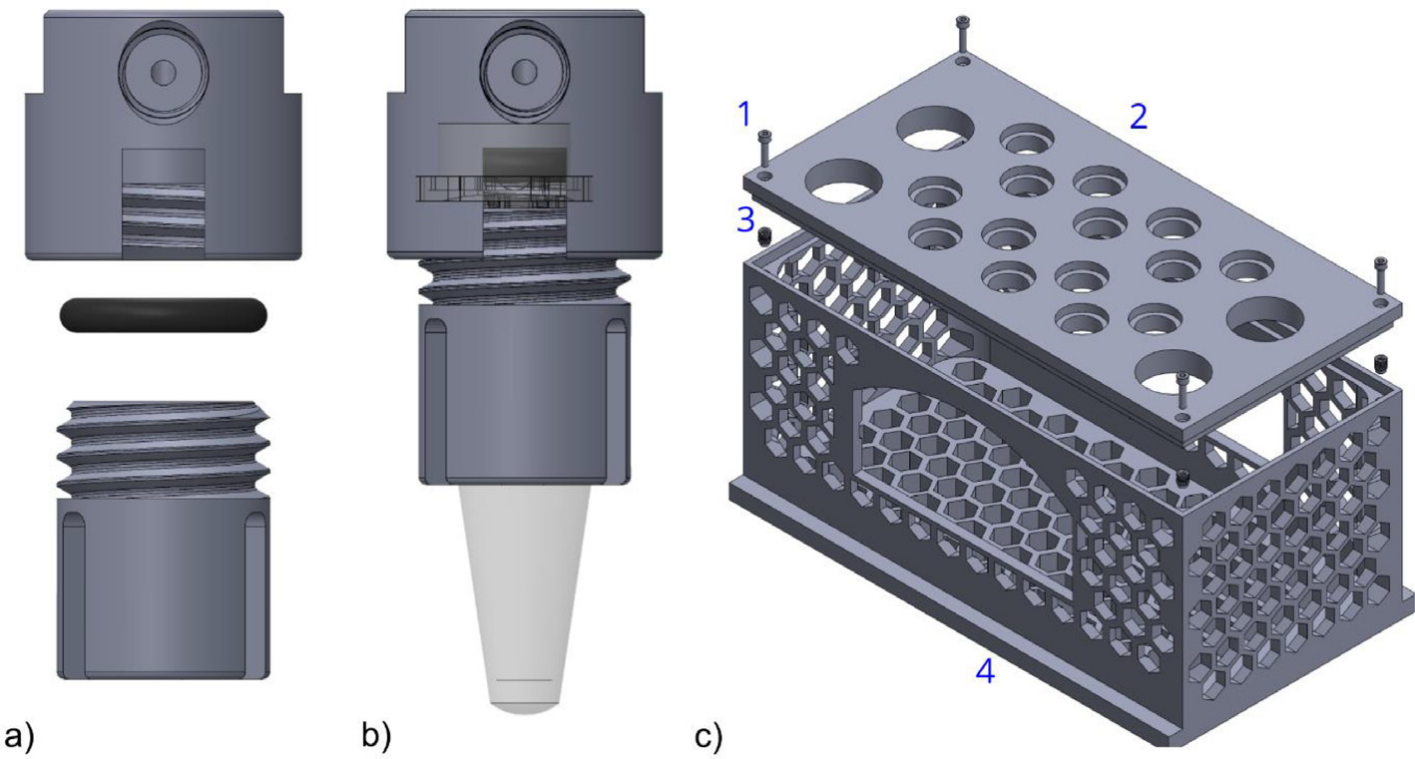
a) Exploded view of the reservoir adapters (A) upper and lower part with an O-ring in between. b) Adapter fitted with 1.5 mL Eppendorf tube. c) Holder (H) design: 1. M3 screw; 2. holder’s cover with holes for sample (small) and waste (large) reservoirs; 3. M3 thread inserts; 4. holder’s body to ensure a stable setting.

The pressure-controlled microfluidic setup (Fig. 1) is designed to automize the sample preparation for single-molecule fluorescence experiments. An external air source with a pressure of *p* = 2.5 bar is connected to the pressure controller D1 which enables to set the pressure from 0 to 2 bar within the microfluidic tubing system. A manifold is connected to the output of the pressure controller D1 to distribute the pressure on up to nine different sample reservoirs, *i.e*. micro reaction tubes (Fig. 2). An adapter A is designed to connect the pressure tube with the reservoir and thus the liquid outlet of the microfluidic tubing system. A holder H allows to arrange the reservoirs. Both adapter and holder were 3D printed. The design is presented in Fig. 3. We used a FDM and SLA printer for comparison. In particular, we printed the adapter with different materials, PSA and stainless steel. The reservoir adapter is designed for *V* = 1.5 mL micro reaction tubes, *e.g.,* Eppendorf tubes, but can be easily adapted to other sizes. The adapter contains two M6 ports to connect with the 1/4“-28 swivel barbed adapter for the pressure inlet and the 1/4”-28 flangeless fittings for the liquid outlet. To ensure the adapter’s airtightness under pressure, an additional flanges gasket metric O-ring is used for the connection of the upper and lower part of the adapter (compare Fig. 3a). A complete single-molecule preparation procedure requires multiple buffer solutions. Therefore, it is efficient to organize all reservoirs within one holder.

There are various types of racks available. Most of them are designed to maximize the number of tubes/sample reservoirs that can be held. In contrast, the here presented holder has a replaceable rack, which can be adapted to various tube sizes. Further, it is designed to guarantee enough space between the individual tubes for their connection to the tubing system. The four-sided honeycomb mesh structured base allows to check the amount of liquid remaining in each sample reservoir and most importantly a cost and time-efficient additive manufacturing. In its present form we use a rack which allows to place fourteen 1.5 mL micro reaction tubes (*e.g.* Eppendorf) and four 50 mL tubes (*e.g.* Falcon, compare Fig. 3).

The software-controlled MUX Wire Valve Controller D2 controls the MUX Distribution valve D3 which combines up to nine individual reservoirs into one tube. We installed a bubble trap D4 to remove potential air bubbles from the tubing system [37]. The microfluidic flow sensor D5 enables the delivery of exact volumes or volume flow rates, respectively, and has a measuring range of 0 up to 1000 µL/min. The pressure controller D1 receives feedback from the flow sensor and allows accurate volume delivery (compare Fig. 5).

The software-controlled MUX Wire Valve Controller D2 controls the 3/2 valve D6. One output connects to the flow chamber F attached to a TIRF or confocal microscope, and the other to the waste reservoir. Thus, unwanted or excess solution in the tubing system is directed to the waste reservoir. The 3/2 valve allows the cleaning of the tubes without involving the flow chamber, which is of particular interest to reduce any contamination of the flow chamber.

We have characterized two flow chamber designs, Grace Bio-Labs HybriWell chamber (dark blue, colour code remains throughout the whole manuscript) and Ibidi µ-slide (dark red), which are both easy to connect to the tubing system and well established in the single-molecule community.

## 3 Design files summary

• Schematic of the hardware assembly: Design of the hardware setup.
• 3D-printed adapter part 1: The upper part of the adapter for the 1.5 mL sample reservoir. It contains two M6 ports to connect the pressure air inlet and liquid outlet tubes. Design adapted from: https://www.elveflow.com/microfluidic-products/microfluidics-accessories/reservoirs/
• 3D-printed adapter part 2: The lower part of the adapter for the 1.5 mL sample reservoir. This part can be screwed tight with the upper part 1 of the adapter to fix the sample reservoir.
• 3D-printed holder part 1: The lid of the holder has different hole sizes for plugging different reservoir sizes (1.5 mL micro reaction tubes and 50 mL tubes).
• 3D-printed holder part 2: The body of the holder with meshed structure.
• Grace Bio-Labs HybriWell chamber CAD for flow simulations: Design and simulation files of the HybriWell chamber with coverslip and press fit tubing connector.
• Ibidi µ-slide chamber CAD for flow simulations: Design and simulation files of the Ibidi µ-slide chamber.
• Software sequence for biotin-BSA passivation procedure: The sequence for automatic single-molecule preparation using ESI (ElveFlow Smart Interface) software from ElveFlow.
• Software sequence for biotin-PEG-passivated chambers procedure: The sequence for automatic single-molecule preparation using ESI for PEG passivated chambers.

## 4 Bill of materials summary

The material summary can be found in Table 2.

**Table 2:** Materials and components summary. Costs for various buffers and chemicals needed for the single-molecule experiments are not included in the total price.

Desig-nator*	Component	Num- ber#	Cost per unit	Total cost	Source of materials	Material type
D1	OB1 Base MK3+with 1 OB1 MKIII CHANNEL 2000	1	3100 €	3100€	https://darwin-microfluidics.com/products/ob1-mk3-flow-controller	n.a.
	KIT MANIFOLD LARGE	1	142 €	142 €	https://darwin-microfluidics.com/products/multiport-manifold-large-kit	n.a.
D2	MUX Wire Valve Controller V2	1	1800 €	1800 €	https://darwin-microfluidics.com/products/microfluidic-valve-controller-pack?variant=14824844787757	n.a.
D3	MUX Distribution Valve	1	2390 €	2390 €	https://darwin-microfluidics.com/products/microfluidic-flow-switch-distributor?variant=37053896229028	n.a.
D4	BUBBLE TRAP M	1	152 €	152 €	https://darwin-microfluidics.com/products/autoclavable-bubble-trap-for-microfluidics?variant=37054418223268	PEEK
D5	Flow sensor 4 Digital	1	1550 €	1550 €	https://darwin-microfluidics.com/products/microfluidic-thermal-flow-sensor?variant=36067511105	n.a.
D6	Controllable Microfluidic 3/2 Port Solenoid Valve	1	299 €	299 €	https://darwin-microfluidics.com/products/remote-controlled-microfluidic-peek-valves?variant=38356954177	n.a.
	Kit Fittings Starter Pack.^1^	1	660 €	660 €	https://darwin-microfluidics.com/products/kit-fittings-starter-pack-luer	PEEKPTFEETFE
	Flangeless Fittings + Ferrules 1/4“-28 to 1/16” OD	1	63 €	63 €	https://darwin-microfluidics.com/products/flangeless-peek-fittings-etfe-ferrules-1-4-28-to-1-16-od-pack-of-10?variant=39491351085220	PEEK and ETFE
	Press Fit Tubing Connectors (pack of 20)	1	27 €	27 €	https://darwin-microfluidics.com/products/press-fit-tubing-connectors-pack-of-20	Silicone
	1/4“-28 Swivel Barbed Adapter Tubing 3/32” I.D. (pack of 10)	1	58 €	58 €	https://darwin-microfluidics.com/products/1-4-28-swivel-barbed-adapter-pack-of-10?variant=31609771720840	Polypropylene
	Ibidi µ-Slide I Luer	1			https://ibidi.com/channel-slides/50--slide-i-luer.html	Polymer
	Grace Bio-Labs HybriWell Sealing System (pack of 100)	100	2.45 $	245 $	https://gracebio.com/product/HybriWell-sealing-system-611102/	Polycarbonate
	Flanges Gasket metric O-ring 15 mm O.D., 10 mm I.D., 2.5 mm wide (pack of 10)	1	7 €	7 €	https://www.amazon.de/10-PCS-Mechanische-Gummi-Ring-Dichtungen/dp/B00CFNN4LC?ref_=ast_sto_dp	Nitrile rubber
H	Reservoir holder^2^	1			n.a.	PLA
A	Reservoir adapter^2^	9			n.a.	PTR, PLA and 17-4Ph stainless steel
	M3 screw and thread	4	<0.25 €	1 €	Local shop	Stainless steel
	Cover glasses / coverslip thickness 1, 24 x 24 mm, n=1.523 (pack of 1000)	1000	<0.03 €	27 €	https://www.carlroth.com/de/de/deckglaeser/deckglaeser-staerke-1/p/h875.2	Borosilicate glass (BK7)

*Numbering according to Fig. 1

# The number was adapted to one experiment.

1 Flow resistances 1/16 OD Tubing 100 µm ID x20 cm; Set of fitting adaptors for connection with pressure source to pressure controller; 1/4”-28 Thread to 3/32“ OD bard x10; male Luer Integral Lock to 3/32“ OD Bard x10; OD PU Tubing 5 m; Flangeless nuts 1/4”-28 Fitting for 1/16“ Tubing with Ferrules x10; Teflon Tubing Spare Roll 1/16” OD x 1/32“ ID 50 m.

2 Material for additive manufacturing: Holder H consumed 1 kg of PLA (30 € per kg) and adapter A consumed 200 mL of Prusament Tough Resin (PTR, 79 € per L). The adapter A was also 3D printed of stainless steel which costs between 50€/kg and 500€/kg. The 17-4Ph used here is about 250€/kg, but only a few grams are needed because the rest of the powder material is recyclable.

## 5 Build instructions

**Pressure-controlled microfluidic system.** The entire microfluidic system is pressure controlled (Fig. 1). It has been built upon the ElveFlow pressure controller system (components are listed in Table 2), but can be adapted to any pressure controller system for microfluidics. The pressure controller D1 requires a stable external source of dry compressed air of at least 2.5 bar = 2500 hPa. There is a port located at the rear of the pressure controller that can be connected to an external pressure source compatible with a 6 mm outer diameter tube. To ensure dry air an additional moisture filter valve is used. On the front of the pressure controller D1 there are different output channels (1 - 4) (Fig. 2a). The 0 to 2 bar channel 1 is used and connected to a 3/32“ internal diameter tube utilizing a self-contained hydrophobic filter to prevent for contamination of the reservoirs and a male luer integral lock ring to bard. A nine-port manifold connects the pressure controller with up to nine sample reservoirs via a swivel barbed adapter and a 3/32” internal diameter tube [38], [38], [39].

**3D printed adapter and sample holder.** To connect the sample reservoirs to the whole microfluidic tubing system, we made up an adapter (A) for 1.5 mL micro reactions tubes (e.g. Eppendorf, Germany) which can be easily modified to different sizes, such as 0.5 mL or 2 mL depending on the individual needs (Fig. 3a). We used two different additive manufacturing methods for comparison: The commonly used stereolithography (SLA) and the novel approach micro selective laser melting (Micro-SLM). In brief, SLA is an additive manufacturing process in which structures are cured from a liquid phase by UV radiation via photopolymerization using as few support structures as possible [40], [41]. Micro-SLM is an additive manufacturing process which irradiates high-flux laser radiation in a powder bed of micro powders with grain sizes smaller than 10 µm and thus fuses them locally. After fusion, the build platform is lowered, powder is reapplied and the powder is irradiated again. In this way, components can be produced layer by layer [40], [41]. The micro-SLM and SLA method have a similar resolution limit in the micrometer range. However, only micro-SLM enables the additive manufacturing of metallic components (e.g. stainless steel) resulting in a broader scope of applications. To ensure airtightness, an O-ring needs to be embedded in the upper part of the adapter (Fig. 3a and Fig. 3b). The M6 port in the front of the upper part is used to connect the 1/4“-28 swivel barbed adapter for the compressed air inlet. The port on top of the upper part is used to connect the flangeless fittings for the liquid outlet. To ensure maximum buffer utilization in the reservoir and to avoid air bubbles in the tube, the liquid output tube needs to be inserted as deep as possible into the reservoir without touching its bottom. Each reservoir adapter is connected to a different channel of the MUX Distribution valve D3.

The reservoir holder is made for fourteen 1.5 mL and four 50 mL tubes and consists of two parts. The upper part 1 is a rack for inserting the tubes and the lower part 2 is the main body including the base of the holder. The two parts are secured by four M3 screws and four M3 threads, respectively (Fig. 3c). The reservoir holder H was designed for fused deposition modelling (FDM), an additive manufacturing process for various strained polymers of low-cost and sufficiently large installation space for the holder. The holder structure is built up layer by layer on a building platform utilizing a heated extruder nozzle [40]. By adding hexagonal structures to the main body, the amount of printing material was reduced without disturbing the structural integrity of the whole holder.

**Flow sensor and bubble trap.** To ensure a constant flow rate in the microfluidic system and to deliver exact pre-defined volumes to the sample chamber, we used a flow sensor. The flow sensor 4 (D5) is directly connected to the pressure controller D1. The feedback from the flow sensor 4 is used to regulate the pressure at the output to allow constant volume flow rates [39], [42].

**Flow chamber.** There are two flow chamber designs used herein, the Ibidi µ-slide [30] and the Grace Bio-Labs HybriWell adhesively attached to a standard #1 BK7 coverslip [31]. Both chambers can be used for objective-based TIRF and confocal microscopy. Whether the chambers can be used for prism-based TIRF has to be shown. The connection of the tube with the µ-slide was achieved using particular adapters (Fig. 2b) [30]. The connection of the tube with the HybriWell chamber was achieved using press-fitting adhesively attached to the HybriWell chamber cover (Fig. 2c). To insert the tube into the press-fitting, the end of the tube needs to be cut at an angle for approximately 3 mm.

**Connection of all devices.** The different devices (A, D3 to D6, flow chamber) were connected by tubes with 1/16“ outer and 1/32” inner diameter, expect $L_{2}$ (length *l*, see Table 3). Therefore, the tubes are pressed into the flangeless fitting and ferrules (Fig. 2b) [40] and connected to the individual devices according to Fig. 1. An important parameter of the whole system is the internal “dead” volume of each component $V_{t1..4}$ (manufacture information) and each tube $V_{1\cdots 5}=\frac{\pi }{4}d^{2}l$. The total dead volume plus the chamber volume $V_{c}$ determines the minimal total buffer volume for each reservoir [37], [42], [43], [44]. Thus, the connecting tubes in the microfluidic system were made as short as possible. The tube lengths *l* and diameters *d* (compare Fig. 1) are listed in Table 3. The volume of each chamber is equal to $V_{c}=A\;h$ with *A* as the surface and *h* as the height of each chamber.

**Table 3:** Summary of all device parameters in the microfluidic setup. Delay time $t_{d}$, maximum pressure $p_{max}$, internal “dead” volume $V_{t1..4}$ of all devices; tube length l, internal tube diameter d, and internal “dead” volume $V_{1\cdots 5}$ of all tubes, base area A, height h and internal volume $V_{c}$ of different flow chambers [30], [31], [37], [42], [43], [44].

Devices	$t_{d}$(ms)	$p_{max}$(bar)	$V_{t1..4}$(µL)
D3: MUX Distribution Valve	280	9	11.6
D4: Bubble Trap M	n.a.	2	95
D5: MFS 4 Flow Sensor	n.a.	15	25
D6: 3/2 Valve	20	2.5	20
**Tubes**	l**(mm)**	d**(mm)**	$V_{1\cdots 5}$**(µL)**
$L_{1}$	400	0.79	196.07
$L_{2}$	209	0.25	10.26
$L_{3}$	100	0.79	49.02
$L_{4}$	100	0.79	49.02
$L_{5}$	250	0.79	122.54
**Chamber**	A**(mm^2^)**	h**(mm)**	$V_{c}$**(µL)**
HybriWell	314	0.15	47
µ-slide	250	0.2	50

**Control software ESI.** The pressure controller, the MUX Distribution Valve, and the MUX Wire Controller were all connected via USB to the workstation and configured by the control software [36]. The MUX Wire Controller was connected via USB to the 3/2 valve to access the control of the gateway. The 3/2 valve has one inlet and two outlet gateways. One of the outlet gateways is normally opened and the other is normally closed [44]. The normally opened gateway is connected to the waste reservoir and the other is connected to the flow chamber, respectively. The control software “ESI” allows for a sequential channel selection and a precise flow rate $\dot{V}$ and pressure *p* control, respectively. The software offers a variety of features, including customizable sequence protocols (SI Fig. 2) as well as the real-time monitoring of experimental parameters within the microfluidic system (flow rate and pressure). This allows for fully automated single-molecule experiments, where the precise control of volume flow rates and the sequence of the respective buffers during the sample preparation is important [45].

## 6 Operation instructions

First, one needs to check whether the external compressed air source is opened and connected to the pressure controller. Second, the control software “ESI” needs to show whether all devices are online in the control interface [36]. To ensure a clean system upon start, the system needs to be flushed with deionized water, followed by isopropanol, and finally dry air to remove all remaining liquids.

**Buffer preparation** Prepare all buffers according to the protocol in the supporting information. Single-molecule fluorescence studies are prone to fluorescence contamination. Thus, all buffers are made with great care under clean conditions. The coverslip used for the HybriWell flow chamber needs to be cleaned before assembling the flow chamber. Rinse the coverslip with DI water. To remove any fluorescence contamination, we recommend placing the coverslip in a plasma cleaner for at least 10 min in low pressure (0.1 bar) oxygen atmosphere [15], [16]. If fluorescence contamination is encountered in single-molecule videos (TIRF) or single-molecule trajectories (confocal), *e.g.,* as high background or single-spots without immobilizing fluorescence labelled biomolecules, restart the procedure as described always starting from the buffer preparation. One advantage of the automated sample preparation is, that each step, *i.e.*, each buffer reservoir, can be validated by taking automated control videos or trajectories for each step to show in which step potential fluorescence contamination appears. All prepared buffers are injected into the micro reaction tubes within the reservoir adapter A, sequentially ordered in the holder H, and connected to channels *No. 1 to n* according to Table 4.

**Table 4:** Order of buffer n for both the sample preparation after biotin-BSA passivation and biotin-PEG passivation (not performed within the microfluidic system), chamber volume $V_{b}$, incubation time $t_{i}$, minimum required buffer volume $V_{r}$, and total injection operation time $t_{n}$ per step.

No. *n*	solution/buffer	$V_{b}$(μL)	$t_{i}$(min)	$V_{r}$(μL)	$t_{n}$(s)
BSA passivation and immobilization					
1	T50	100	0	1351.8	26.71
2	Biotin-BSA:BSA solution	50	10	819.38	20.71
1	T50	100	0	1351.80	26.71
3	Neutravidin in T50 buffer	100	5	869.38	26.71
4	1xSB	100	0	1351.80	26.71
5	5'-biotin-Cy3/5-DNA sample solution in 1xSB	50	5	819.38	20.71
4	1xSB	100	0	1351.80	26.71
6	IB50	200	5*	969.38	38.71
**PEG passivation**^1^**and immobilization**					
1	T50	100	0	869.38	26.71
2	Neutravidin in T50 buffer	100	5	869.38	26.71
3	1xSB	100	0	1351.80	26.71
4	5'-biotin-Cy3/5-DNA sample solution in 1x SB	50	5	819.38	20.71
3	1xSB	100	0	1351.80	26.71
5	IB50	200	5*	969.38	38.71

*Incubation time before imaging.

1 The PEG passivation, i.e., PEGylation procedure, is explained in the SI.

**Fig. 4:**
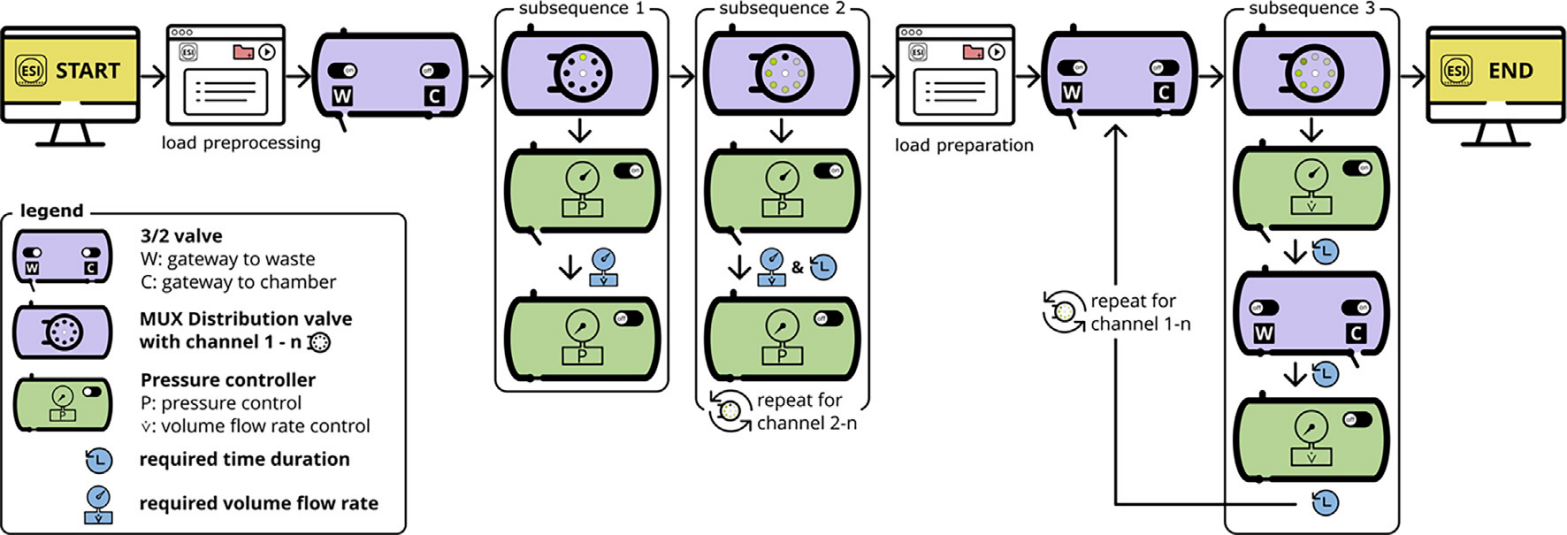
Flow chart of the hardware control sequence within the software ESI. The pre-processing sequence, i.e., the initialization of the microfluidic system, including subsequences 1 and 2 to fill the whole tubing system and to remove any air bubbles, is followed by the preparation sequence, i.e., the actual flow chamber preparation for single-molecule experiments, including subsequence 3. Find subsequences 1-3 in the SI Fig. 9.

**Automated sample preparation** We follow a standard protocol for single-molecule fluorescence experiments (SI Fig. 1), starting with 1) the surface passivation either with biotin-BSA or biotin-PEG, followed by 2) the sample immobilization and 3) adapting the imaging buffer conditions. The procedure is exemplified for a standard duplex forming Cy3 and Cy5 carrying 44nt long DNA oligonucleotide with a 5‘-end biotin functionalization for surface immobilization. In the case of a one-spot confocal experiment for fluorescently labelled biomolecules freely diffusing in solution, the immobilization procedure is redundant. The flow chart in Fig. 4 describes the program workflow for the automated sample preparation. The program consists of two main processes: preprocessing and sample preparation. The latter process is configured for each of the two single-molecule immobilization methods presented herein. To start the pressure-controlled microfluidic system, download the zip file from the repository and unzip the file (https://osf.io/r8enm/). The starting panel in the microfluidic control software allows the opening of a sequence interface and the loading of the desired program. To start the automated single-molecule preparation first load and run the *main.sq* file in the preprocessing folder and second load and run the *main.sq* file in the preparation folder [36], [45].

**Preprocessing.** First, the air in the whole system needs to be removed starting with the tube length *L*_1_ for buffer reservoir *No. 1* (compare nomenclature in Fig. 1). As the flow sensor D5 only operates with liquid in the tube system, the pressure controller is set to a constant value of 200 mbar (Fig. 4, preprocessing procedure with subsequence 1, sequence details in SI Fig. 2). Each buffer is now pressed from the reservoir to the flow sensor D5. As soon as the flow sensor detects the liquid, *i.e.*, showing a flow rate $\dot{V}$, the MUX Distribution valve D3 changes to the next channel *No. n + 1* (Fig. 4, preprocessing procedure with subsequence 2, sequence details in SI Fig. 2). The total preprocessing buffer volume $V_{v}$ for each channel is $V_{v}=\sum_{i=1}^{3}V_{i}+V_{\mathrm{ti}}=387\;\mu L$. From the second channel onwards, the remaining previous buffer between the MUX Distribution valve D3 and the flow sensor D5 should be completely pressed to the waste reservoir via the 3/2 valve D6. The necessary time $t_{v}$ for each channel has been experimentally determined to be at least 5 s per channel.

**Sample preparation.** According to the chosen passivation method, the buffer of each step *No. 1* to *n* sequentially passes through the microfluidic system to be eventually injected into the flow chamber (Table 4 and Fig. 4, preparation procedure with subsequence 3, sequence details in SI Fig. 2). The remaining buffer volume $V_{w}=\sum_{i=1,j=2}^{4}V_{j}+V_{\mathrm{ti}}=260\;\mu L$ between the MUX Distribution valve D3 and the 3/2 valve D6 of the respective previous step needs to be removed. Here, the flow sensor D5 is used to achieve a stable flow rate of $\dot{V}=500\mu L/min$ and the time required to remove the volume $V_{w}$ is $t_{w}=\frac{V_{w}\;\;}{\dot{V}}=\;31.19\;s$.

The respective buffer volume $V_{r}$ in each reservoir should not be less than the sum of all volumes $V_{r}=V_{v}+{n(V}_{w}+V_{5}+V_{b})$ for the preprocessing $V_{v}$, the sample preparation including the dead volume $V_{w}$, the dead volume $V_{5}$ of the tube length $L_{5}$ after the 3/2 valve D6 and the actual volume of the flow chamber $V_{b}$. The time required for each channel to inject the desired buffer volume $V_{b}$ is equal to $t_{n}=\frac{V_{5}+V_{b}}{\dot{V}}$ (Table 3).

## 7 Validation and characterization

To ensure a successful automated sample preparation, the flow velocities *v* in the flow chamber must be rather low to keep the passivation and immobilization intact. The theoretical flow velocities are determined as a function of the applied pressure *p* and using flow simulations (*vide infra*).

**Fig. 5:**
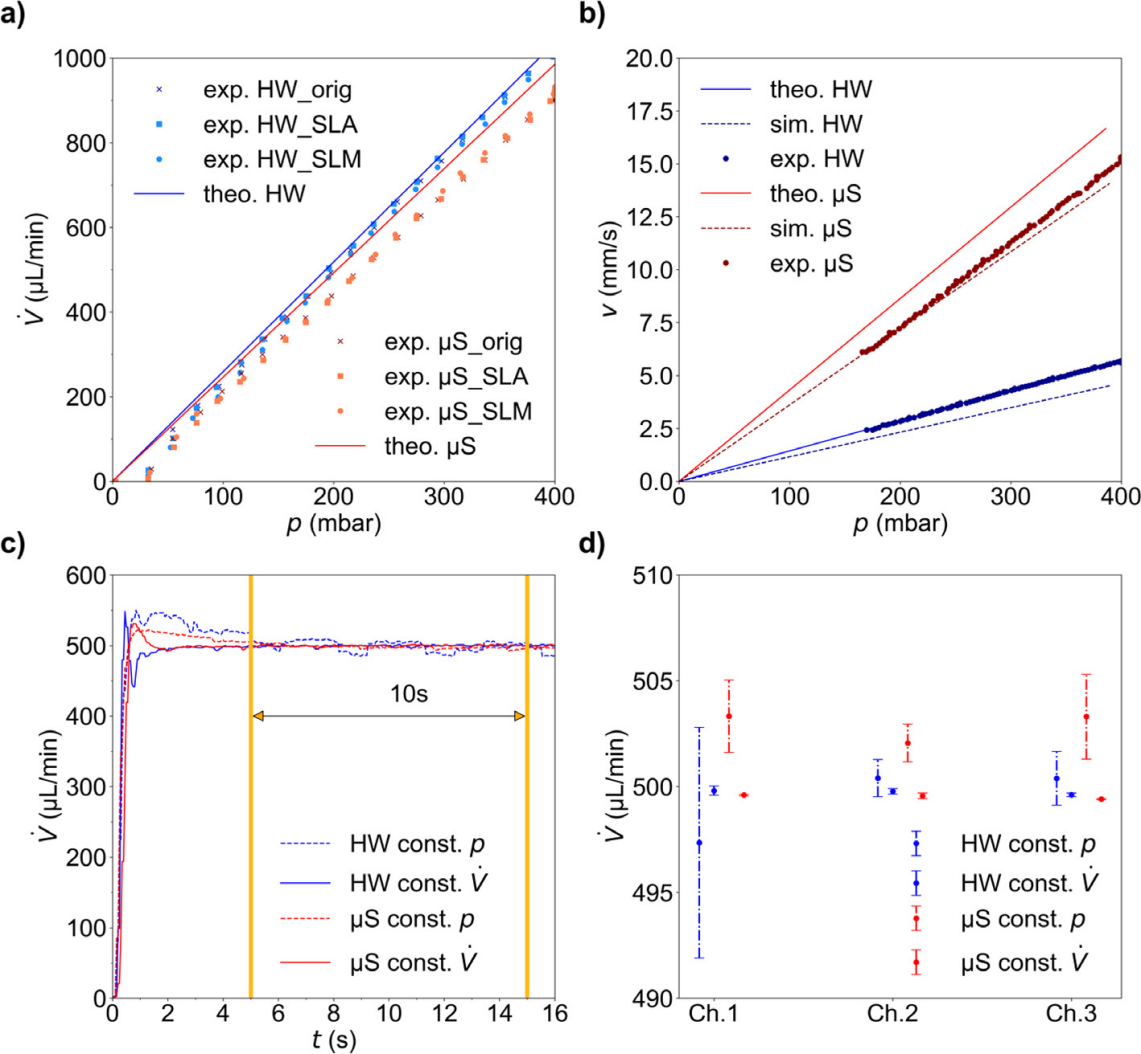
a) Experimentally determined and theoretical volume flow rates as a function of the applied pressure for different reservoir adapters A: commercially available adapter for comparison (ElveFlow, org) and 3D printed adapters for both SLA and SLM used in combination with both chambers, Ibidi µ-slide (µS) in red and Grace Bio-Labs HybriWell (HW) in blue. b) Comparison of the simulated, theoretically calculated and experimentally determined flow velocities as a function of the applied pressure for both chambers. c) Experimentally determined volume flow rates in the exemplarily chosen channel *No. 2* for both chambers as a function of time with a constant pressure set by the pressure controller (dashed) and a constant volume flow rate set to 500 µL/min (solid) controlled by the flow sensor. d) Mean value and standard deviation of the volume flow rate in c) for channels *No. 1* to *3* at a given volume flow rate $\dot{V}$ = 500 µL and a constant pressure (manually set by the pressure controller). The measurements were taken three times for each channel starting at 5 s to 15 s (orange interval in c).

With the continuity equation $\overset{˙}{V}=\frac{\mathrm{dV}}{\mathrm{dt}}=v\cdot A=const.$. the flow velocity *v* is determined by the cross-sectional area *A* of the chamber or tube (Table 3) and a specified volume flow rate $\dot{V}$ which can be set with the flow sensor D5. According to Hagen-Poiseuille’s law $\dot{V}=\frac{1}{R}\cdot \Delta p$ the volume flow rate $\dot{V}$ follows a pressure gradient $\Delta p=p_{1}-p_{2}$ given the resistance $R=\frac{8\cdot \eta \cdot l}{\pi \cdot {\left(d/2\right)}^{4}}$ with the viscosity of water η at 20°C, the tube diameter *d* and tube length *l* in a laminar flow scenario. Within our tubing system the total resistance ${R=R}_{1}+R_{2}+\cdots +R_{i}$ was calculated given the parameters in Table 3, but the total resistance *R* is mainly determined by the flow resistance tube in front of the flow chamber (Table 2). This resistance tube has a very small inner diameter with a 100-fold higher resistance compared to all other components. This is necessary to ensure a stable pressure control range. The theoretical volume flow rate and the flow velocity within the chamber in dependency on the applied pressure is shown in Fig. 5a and Fig. 5b.

**Fig. 6:**
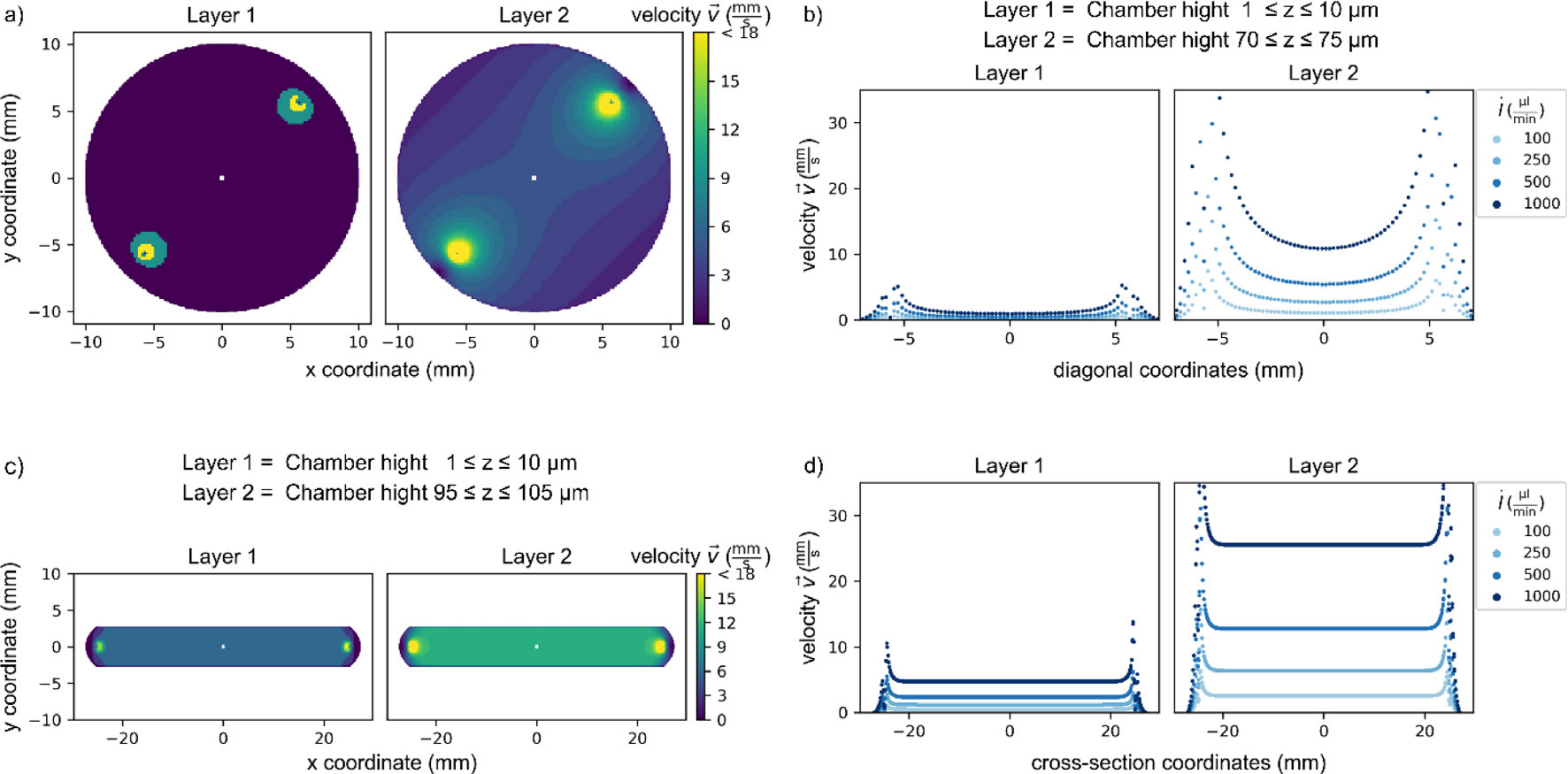
Flow velocities for the Grace Bio-Labs HybriWell (a, b) and Ibidi µ-slide (c, d) flow chamber layouts exemplified for different heights *z* of the horizontal cross section. (a, c) 2d-velocity distribution close to the surface (Layer 1) and at half height of the chamber (Layer 2). (b, d) Flow velocities for sectional view between inlet and outlet openings for different volume flow rates $\dot{V}=\;100,\;250,\;500,\;1000\;\mu l/\min$.

We measured the volume flow rate with the flow sensor D5 increasing the pressure for different 3D printed reservoir adapters and the original adapter from ElveFlow for both sample chamber designs and compared it with the theoretical values (see Fig. 5a). All adapters show the same $p-\dot{V}$ dependency and the deviations from the theoretical values are negligible. The required pressure for the experiment is around 200 mbar, thus, all tested reservoir adapters can be used to guarantee air tightness and a functional microfluidic system. For comparison, we show the experimentally (flow sensor), theoretically and via flow simulations (*vide infra*, Fig. 6) determined p-v dependency in Fig. 5b. In both cases, the experimental flow velocity values lie between the simulated and theoretical values. The flow velocity within the narrow, rectangular µ-slide is larger than in the wide, round HybriWell chamber which is expected. The theoretical laminar flow scenario in a tube of known length and diameter corresponds best with the experimental values in the HybriWell chamber. The µ-slide, however, is best described by the flow simulation taking the actual boundary conditions and chamber geometry into account.

Setting the volume flow rate via the flow sensor requires two ESI software-specific parameters P and I, both of which have a range from 0.001 to 1 [36], [42]. P affects the pressure regulation to be stable or fast. I affects the regulation to be smooth or responsive. By using different values and a combination of P and I (compare SI Table 1), the volume flow rate regulation was optimal at a setting of 0.05 for both parameters (Fig. 5c). Interestingly, the volume flow rate also depends on the flow chamber design. Compared to the Grace Bio-Labs HybriWell chamber with a circular geometry, the flow rate of the Ibidi µ-slide chamber with a rectangular geometry is much smoother.

The pressure in the microfluidic system is set by the pressure controller to a constant value (passive control) or adjusted by setting the desired volume flow rate measured from the flow sensor (active control). We measured the volume flow rates for three different channels *No. 1 to 3* using both chamber designs and pressure methods and calculated the average and standard deviation for a 10 s measurement period (Fig. 5d). Setting the pressure actively by the flow sensor results in a steady volume flow rate in the system for all channels. In contrast, there is a relatively large deviation in the volume flow rate with a constant pressure setting. The reason may be the deviation of the tube length, the different bending of each tube, or potential differences in the MUX Distribution valves for each channel. In any way, we commend to use the constant *p* setting to achieve constant volume flow rates in the microfluidic system.

To determine the actual volume flow rates and flow velocities within the flow chamber, flow simulations were carried out with the software SolidWorks at different volume flow rates (Fig. 6). Here, the flow velocities close to the imaging surface are of particular interest, to investigate whether the passivation and immobilization of biomolecules stay intact upon flushing the chamber. To set up the flow simulation an adapted CAD file for both flow chambers was created (see Fig. 1 and Table 1). The flow simulation was carried out for different volume flow rates and a laminar flow of water at T=293.2K (Table 5). The chamber walls were presumed to be adiabatic with a roughness of 10 μm. In this project, the “static pressure mean value” and “velocity mean value” were chosen for the inlet opening of the chamber, and the “volume flow” and “velocity mean value” for the outlet opening of the chamber since flow velocity and volume flow rate must satisfy the continuity equation. For the calculation of the chamber resistance, the static pressure at the inlet is required. After entering the boundary conditions, the simulation grid was generated according to the local and global network coordinates found in SI Table 1 and the simulation was started.

**Table 5:** This table contains the boundary conditions for the flow simulation. The outlet pressure was calculated for a given volume flow rate and the resistance of the final tube from the chamber to the waste reservoir with standard atmospheric pressure at T=293.2K.

Volume flow rate $\dot{V}$ in μl/min	Outlet pressure *p* in hPa
100	1013.60
250	1014.12
500	1015.00
1000	1016.76

The flow profile in the Ibidi µ-slide chamber is homogeneous. In contrast, the flow profile of the Grace Bio-Labs HybriWell chamber is more heterogeneous. The flow velocities for both chambers are rather low close to the surface, *i.e*., in the area of the immobilized biomolecules. The HybriWell comprises a flow velocity v<1mm/s in the area of the field of view of the particular imaging device (FOV, white square of approx. 100 × 100 µm^2^ in the centre of both chambers in Fig. 6a and 6c). The flow velocities in the Ibidi µ-slide chamber are v<4mm/s at the targeted volume flow rate of 500 µL/min. Further, we calculate the critical flow velocity $v_{\mathrm{crit}}$ to keep the passivation and immobilization intact, starting with the wall shear stress $\tau_{w}$

$$\tau_{w}=\frac{6\cdot \eta \cdot \dot{V}}{w\cdot H{\left(x\right)}^{2}}$$

with η the viscosity of the medium, w the width of the chamber, and $H\left(x\right)$ the height of the chamber at position x, and applying the continuity equation and assuming $H\left(x\right)=const.=h$, the critical flow velocity $v_{\mathrm{cirt}}$ is

$$v_{\mathrm{crit}}=\frac{\tau_{w_{\mathrm{crit}}}\cdot h}{6\cdot \eta }.$$

With the critical wall shear stress $\tau_{w_{\mathrm{crit}}}=\left(12.6\pm 1.2\right)\;\text{dynes / c}{\text{m}}^{\text{2}}$ where 50% of the initially bound biotin-BSA molecules are still adhering to the surface [46], we reach a critical flow velocity of $v_{\mathrm{crit}}=32\;\text{mm/s}$, which is well above the flow velocities observed in both characterized chambers and the applied volume flow rates. To predict the flowing behaviour within the chamber we first calculate the Reynolds number of the rectangular chamber. Here, the flow velocity v was assumed to be 50 mm/s, the characteristic length l=4.9mm was set to the inner diameter of the rectangular chamber inlets. The according Reynolds number Re=ρ·l·v·η-1 = 245 is well below the critical Reynolds number $Re_{\mathrm{crit}}=2300$ of a turbulent flow. Since the inner diameter of the inlets and outlets of rectangular chamber are even larger than the inner diameter of the round chamber, the Reynolds number of the round chamber will be small too, and turbulent flow is unlikely. However, imaging close to the inlet and outlet is not recommended due to the high flow velocities and a potential disruption of the surface passivation.

## Ethics statements

The work complies with the ethical guideline of HardwareX and did not involve human subjects or animal experiments.

## CRediT authorship contribution statement

**Anxiong Yang:** Methodology, Software, Investigation, Visualization, Writing – original draft. **Falk Nicolas Lein:** Investigation, Formal analysis, Visualization, Writing – original draft. **Joana Weiler:** Methodology, Investigation, Writing – review & editing. **Julian Drechsel:** Methodology, Writing – review & editing. **Vanessa Schumann:** Methodology, Investigation, Writing – review & editing. **Felix Erichson:** Visualization, Writing – review & editing. **André Streek:** Methodology, Supervision. **Richard Börner:** Conceptualization, Methodology, Supervision, Funding acquisition, Writing – original draft, Writing – review & editing.

## Declaration of Competing Interest

The authors declare that they have no known competing financial interests or personal relationships that could have appeared to influence the work reported in this paper.
