# Supplementary material for: Pressure-controlled microfluidics for automated single-molecule sample preparation
Source: HardwareX. 2023 Apr 27;14:e00425. doi: 10.1016/j.ohx.2023.e00425 (PMC10329172; doi:10.1016/j.ohx.2023.e00425)
Supplement: Supplementary Data 1 [file mmc1.pdf]

Supporting Information

Pressure-controlled microfluidics for automated single-molecule sample preparation

Anxiong Yang, Falk Nicolas Lein, Joana Weiler[1], Julian Drechsel, Vanessa Schumann, Felix Erichson, André Streek, Richard Börner*

Laserinstitut Hochschule Mittweida, University of Applied Science Mittweida, Technikumplatz 17, 09648 Mittweida, Germany

1. Present address: Institute of Physics, University of Lübeck, Ratzeburger Allee 180, 23556 Lübeck, Germany

* Corresponding author: richard.boerner@hs-mittweida.de

Abbreviations

- SB standard buffer
- T50 TRIS buffer
- AB50 anti-blinking buffer
- APTES 3-Aminopropyltriethoxysilane
- SAB50 sugar anti-blinking buffer
- OSS oxygen scavenger solution
- IB50 imaging buffer
- meOH methanol
- MOPS 3-N-morpholinopropane sulfonic acid
- RT room temperature
- TRIS Tris(hydroxymethyl)aminomethane
- DI de-ionized
- BSA bovine serum albumin
- PEG polyethylene glycol
- SVA succinimidyl valerate
- Trolox 6-hydroxy-2,5,7,8-tetramethylchroman-2-carboxylic acid

Buffer materials

The amount of each buffer and the required incubation time are listed in Table 4 in the main article and are used to calculate the process parameter for the automated preparation. The materials used for the chamber passivation and the preparation of single-molecule samples are listed below:

- DI H_2O (sterile filtrated)
- neutravidin in T50 buffer (0.2 mg ml^{-1})
- 5X SB
  - 10 mL DI H_2O
  - 0.4 M MOPS, pH 6.9 (use KOH or HCl for pH adjustment)

- sterile filtered and stored at 4 °C in the dark

- T50
  - 10 mL DI H_2O
  - 50 mM TRIS, pH 7.6
  - 50 mM KCl
  - sterile filtered and stored at RT
- AB50 buffer
  - 80 mM MOPS, pH 6.9
  - 500 mM KCl

  → in 2 mL 5X SB
  - add one spatula tip of Trolox
  - add 8 mL DI H_2O
  - sterile filtered and stored at 4°C in the dark
- SAB50 buffer
  - 100 mg mL^{-1} D-Glucose

  → in 1 mL AB50
  - sterile filtered and stored at 4°C in the dark
- OSS
  - 10 µL catalase
  - 3 mg glucose oxidase

  → in 90 µL T50 buffer
  - store on ice
  - optional: add 15 µL mercaptoethanol or saturated 2 mM Trolox solution
- IB50 buffer
  - 2 µL OSS

  → in 198 µL SAB50 buffer
- working solution for the immobilization of fluorescently labelled DNA sample (50 pM) in 1x SB buffer
  `5’-biotin- ATT TAT GGA TGT TCA CT-Cy5-A GTG TCT TCG GAC ACT AGT GAA CAT CC-Cy3 3´`
- Biotin-BSA solution (biotin-labelled BSA and BSA 1:99)
  - 1 mg mL^{-1} biotin-BSA:BSA

  → in T50 buffer
- PEGylation solution
  - 6 mg biotin-PEG-SVA
  - 240 mg PEG-SVA

  → in 1920 µL 0.1 M $NaHCO_3$ buffer, pH 8.5
- aminosilanization solution
  - 100 ml meOH
  - 5 ml acetic acid
  - 3 ml APTES

Surface passivation with PEG

After cleaning the coverslips (#1) with a plasma cleaner (oxygen atmosphere) to remove any fluorescence contamination, incubate the coverslips for 30 min in the aminosilanization solution at room temperature. Rinse the coverslips with methanol and dry them with nitrogen gas. Place the coverslips in a humid environment (e.g. build a humid chamber) and incubate them overnight with 35 µL PEGylation solution. Wash the coverslips after incubation with DI H_2O and dry them with nitrogen gas. Merge the PEG-passivated coverslips with the HybriWells to create the microfluidic chambers.

Sample preparation and immobilisation

Flush the passivated microfluidic chamber with 100 µL T50 buffer. Add 100 µl neutravidin (0.2 mg mL^{-1}) in T50 buffer and incubate the chamber for 5 min. Flush the chamber with 100 µl 1xSB buffer to remove unbound neutravidin. Immobilize 50 µL Biotin-Cy3/5-DNA oligonucleotide in 1xSB and incubate for 5 min. Flush the chamber with 100 µL 1xSB buffer to remove unbound DNA. Add 200 µL IB50 buffer and incubate the chamber for 5 min at room temperature before starting the imaging [15–17].

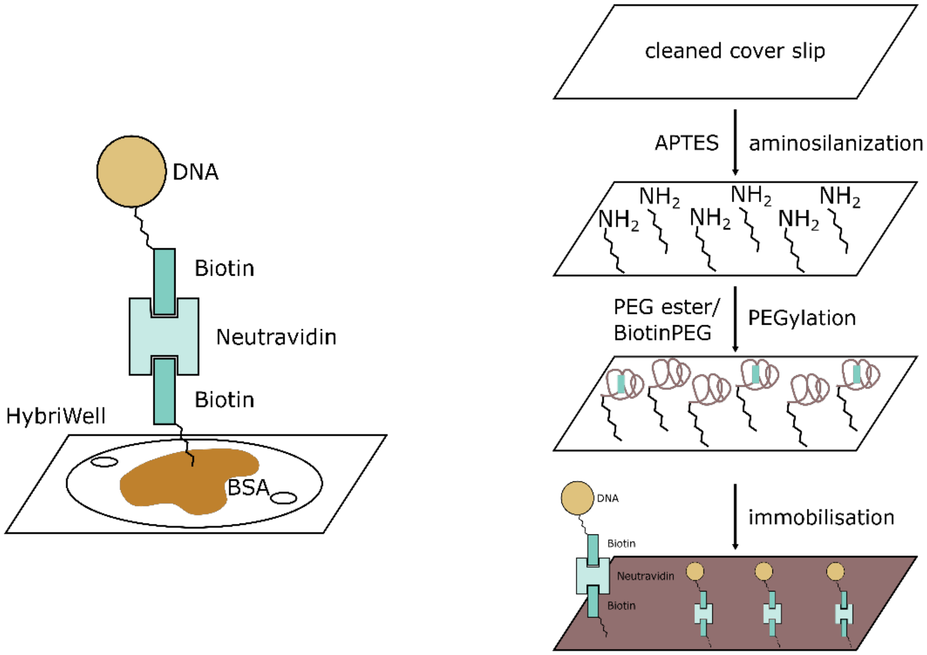

SI Figure 1. Schematic of the immobilization procedures with (left) biotin-BSA and (right) PEG-biotin. The latter includes a schematic of the PEG passivation procedure.

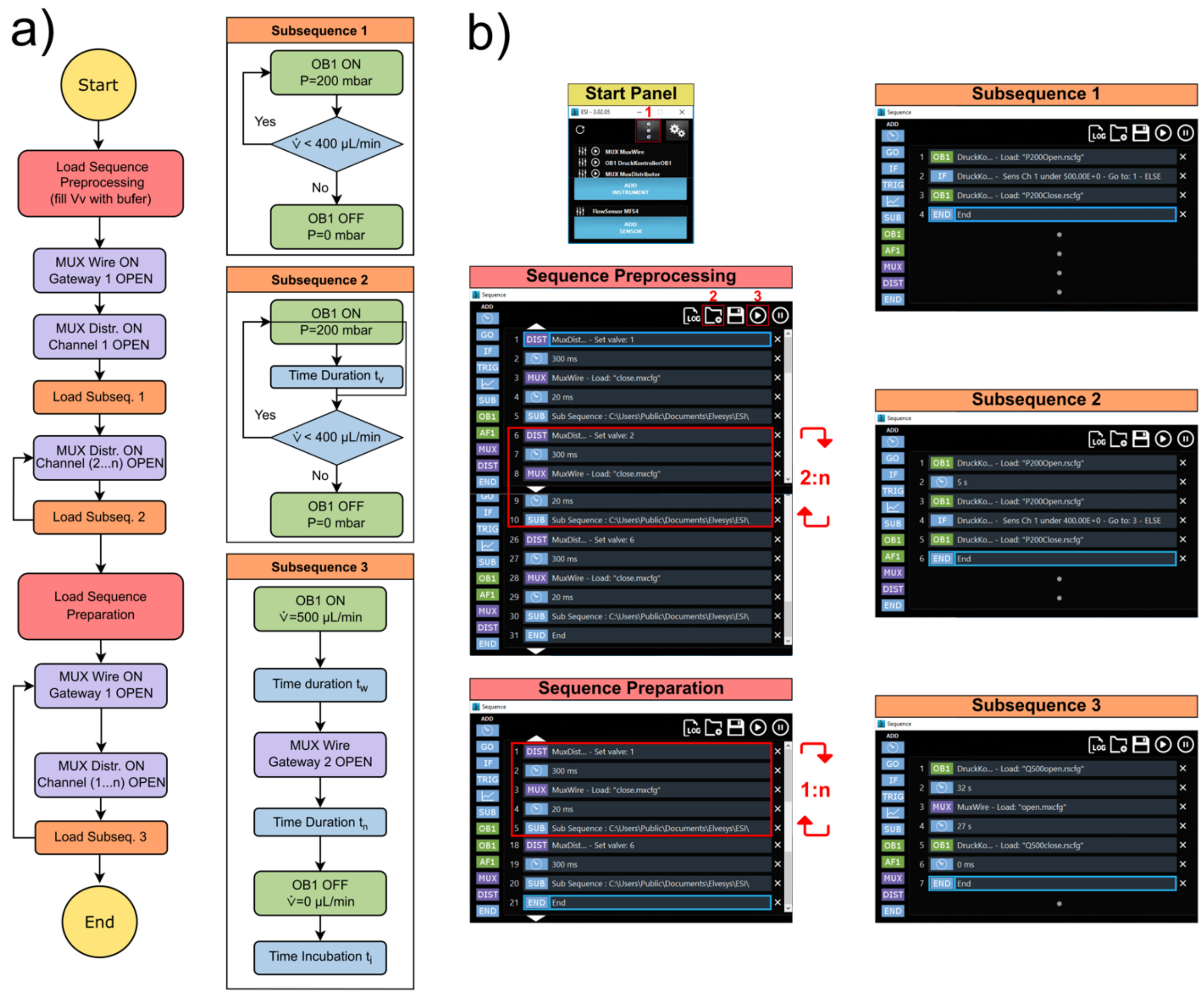

SI Figure 2. a) Flowchart of the software control sequence. b) The starting panel (yellow) and the sequence/subsequence interface (red and orange) of the microfluidic control software ESI show the program sequence "preprocessing", "preparation" and subsequence 1-3. Press button **1** to open the sequence interface, **2** to load the *main.sq* sequence and **3** to run the loaded sequence. The sample preparation will continue automatically and can be adapted according to individual protocols.

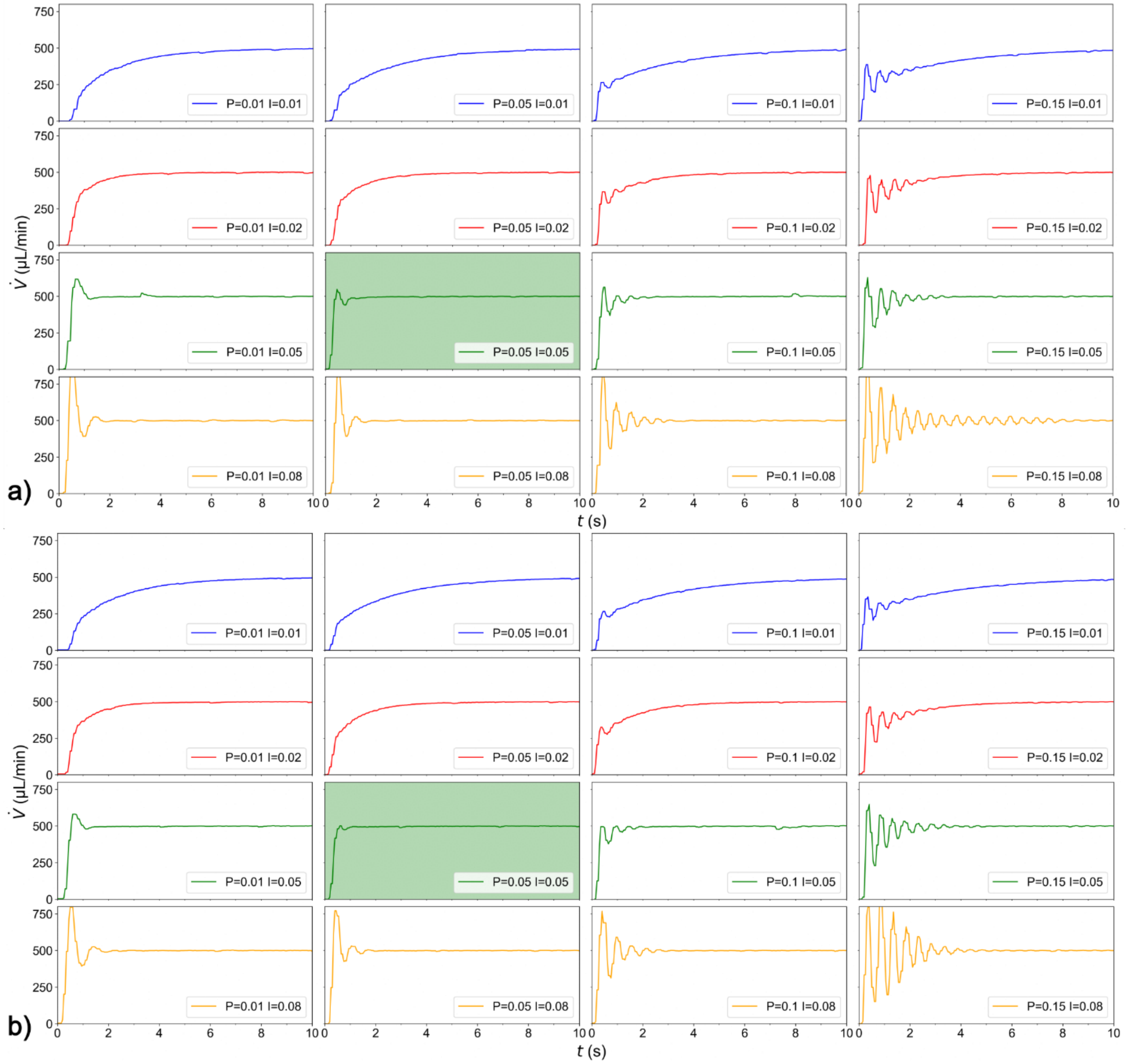

SI Figure 3. Time dependent volume flow rate of a) the Grace Bio-Labs HybriWell chamber and b) the Ibidi µ-slide chamber for different combinations of flow sensor parameters I and P which can be varied in a range from 0.001 to 1. I is the parameter for smooth to responsive and P for stable to fast. The subfigure with green background represents the best parameter combination, comprising a fast but stable increase of the volume flow rate till the desired volume flow rate $\dot{V} = 500\ \frac{\mu L}{min}$ is reached.

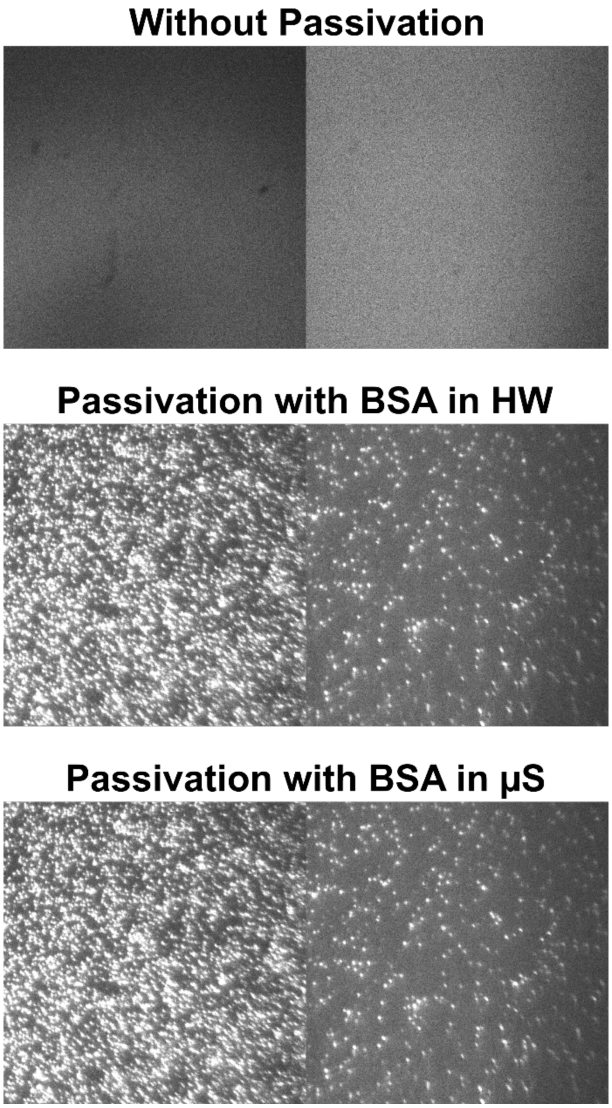

SI Figure 4: Sample TIRF images of thousands of immobilized single-molecules (1 nM Cy3 and Cy5 labelled biotinylated DNA oligonucleotides) with a BSA-biotin passivation in both chambers, Grace Bio-Labs HybriWell and Ibidi μ-slide. A TIRF image of the HybriWell chamber without BSA passivation, but running the complete preprocessing and preparation sequence is shown for comparison.

SI Table 1. Parameter settings for the basic and local simulation area network.

	HybriWell	μ-slide
Global network		
Basic network		
N_x	27	52
N_y	27	30
N_z	60	8
Number of cells	43740	12480
Cell size in mm	0.742 x 0.742 x 0.0346	1.077 x 0.65 x 0.065
Refinements		
Cell refinement	4<br>0	off
Channels	off	off
Advanced mesh refinement	2<br>0<br>Standard<br>1<br>Standard	off
Close small columns	Standard	off
Local network		
Cell refinement	2<br>0	3<br>0
Channels	off	off
Advanced mesh refinement	2<br>0<br>Standard<br>1<br>Standard	2<br>0<br>Standard<br>2<br>Standard
Close small columns	off	off
Number of cells	635542	618122
Cell size in mm	0.1856 x 0.1856 x 0.00865	0.135x 0.0862 x 0.081
